# Pensions for All Mothers

**Published:** 1919-09-20

**Authors:** 


### Maternity and Child Welfare.

### PENSIONS FOR ALL MOTHERS.

In a recent remarkable address Lady Leslie Mackenzie (Edinburgh) stated that we had heard much of the "One Man, One Vote! One Man, One Job!" but little about the "One Woman, One Job!" Was it possible to expect a woman to be a wage earner and a mother too? Of the thousands of mothers and expectant mothers who were found to earn a living, the majority of their babies found their way eventually into institutions, and there became institutionalised. By no cut-and-dried methods could babies be reared. A baby needed more than a bottle and a beautiful crib and attention at stated intervals. It needed cuddling, dandling, the close touch of a warm human body; human love was required for the best development physically and morally. Institutions could not, and did not, give "love." Mother and child should be kept together, married or unmarried, and at whatever cost. Is it cheaper to establish and run day nurseries at the expense of the State, or provision to enable a woman to live in her own home? There was a great need of an alteration in the medical training of men and women concerning the growth and the life of the newly born, and in State-aided institutions there should be no cheeseparing.

Dr. Vinnig emphasised the necessity of the better education of the medical student concerning infant life. In taking the greatest degree in England—the M.D.—the knowledge of infants and infant feeding was less than nothing. Mrs. Palmer, of Southampton, speaking on how to keep mother and child together, suggested a scheme of motherhood pensions, applicable to all classes, and to whosoever applied for it. This should be the State's acknowledgment of a service rendered. Interesting facts and figures were given by Colonel Carless, F.R.C.S., in relation to Dr. Barnardo's Homes.

